# Bivalent IAP antagonists inhibit TRAF2-bound cIAPs and limit TNF-mediated NF-*κ*B signaling

**DOI:** 10.1038/cddis.2016.283

**Published:** 2016-09-29

**Authors:** Stephen M Condon

**Affiliations:** 1 TetraLogic Pharmaceuticals Corporation, 343 Phoenixville Pike, Malvern, PA 19355, USA

Birinapant is a bivalent antagonist of the Inhibitor of Apoptosis (IAP) family of proteins.^1^ IAP antagonists, also termed Second Mitochondria-derived Activator of Caspases (Smac)-mimetics, have emerged as potential treatments for cancer^2, 3, 4, 5, 6^ and infectious disease.^7^

The mammalian IAPs are multi-domain proteins, which contain at least one Baculovirus IAP Repeat (BIR) domain and several IAPs, including the two cellular IAPs (cIAP1 and cIAP2) and the X chromosome-linked IAP (XIAP), contain three functionally-unique BIR domains, that is, BIR1, BIR2, and BIR3. Early reports identified XIAP as an apoptosis suppressor via the direct interaction between caspases and the XIAP BIR3, and BIR2 domains. Endogenous Smac, released from the mitochondria, binds to XIAP through its *N*-terminal tetrapeptide, that is, AVPI, which promotes displacement of the active caspases and further propagation of the apoptotic process. The observation that many cancers had increased XIAP expression led to the hypothesis that small-molecule Smac-mimetics targeted against XIAP might act as pro-apoptotic agents for the treatment of cancer. Although development of IAP antagonists derived from this XIAP/caspase hypothesis, it is now recognized that IAP BIR domains mediate diverse signal transduction pathways by modulating a variety of protein-protein interactions, and that the majority of BIR3 domain-directed IAP antagonists induced cancer cell death by antagonizing the cIAPs with or without XIAP involvement.

cIAP1 and cIAP2 are critical E3 ubiquitin ligases, which are responsible for the signal-induced post-transcriptional modification of multiple proteins at the TNF Receptor 1 (TNFR1), the NLRP3-caspase-1 inflammasome, and B-cell survival and responsiveness. As such, the cIAPs occupy a unique position for regulating cell-cell communication via exogenous ligands (TNF, TRAIL), intracellular response against invading pathogens, and humoral immunity.^3, 8^ As IAP antagonist treatment results in the auto-ubiquitylation of cIAP1, and cIAP2 to various extents, with subsequent loss of the cIAPs via the ubiquitin-proteasome system,^1, 9^ IAP antagonists have the potential to interfere with multiple signaling and regulatory processes including immunomodulation, inflammation, and cancer cell survival.

One challenge for the development of IAP antagonists has been correlating their diverse chemical structures with their effect on these multiple downstream signaling events. Mechanistically, it remains unclear as to whether bivalent IAP antagonists engage only IAP BIR3 domains or both BIR2 and BIR3 domains to exert their biological action.^9^ Similarly, the lack of selectivity amongst IAP family members by bivalent pan-IAP antagonists is reported to predict specific inflammation-mediated toxicities, which could limit their therapeutic application.^10, 11^ Finally, bivalent IAP antagonists but not their monovalent cousins are reported to induce the degradation of the single BIR domain-containing melanoma IAP (ML-IAP), supporting the hypothesis that bivalent IAP antagonists might interact with IAP proteins in ways that are not available to the monovalent species.^12^

In the paper by Mitsuuchi *et al.*,^13^ scientists at TetraLogic Pharmaceuticals describe another consequence of bivalent IAP antagonists treatment – the inhibition of TNFR1-mediated NF-*κ*B activation. Despite the comparable ability of bivalent and monovalent IAP antagonists to deplete cIAP1 with subsequent cancer cell death in sensitive cell lines, only bivalent IAP antagonists, such as birinapant, were able to inhibit the TNF-mediated activation of p65/NF-*κ*B, that is, nuclear translocation of p65/NF-*κ*B and the activation of NF-*κ*B promoter-linked luciferase reporter gene (see Figure 1). The authors have attributed these results, at least in part, to the inability of monovalent IAP antagonists to effectively deplete cIAP1 at the TRAF2 complex. Since functional cIAP1 is maintained within the TNFR1 signaling complex by the cIAP1 BIR1:TRAF2 interaction, failure to deplete a portion of this TRAF2-associated cIAP1 following monovalent IAP antagonist treatment was sufficient to retain NF-*κ*B signaling.

Importantly, despite both classes of IAP antagonists inducing the formation of a pro-apoptotic RIPK1:caspase-8 complex, the authors suggest that the inhibition of p65/NF-*κ*B nuclear translocation by bivalent IAP antagonists only (of >300 bivalent and monovalent IAP antagonists tested) implies a unique property of bivalent IAP antagonists, which might be exploited in other therapeutic areas like infectious disease or in combination with certain immunotherapies. Interestingly, inhibition of nuclear translocation of p65/NF-*κ*B by birinapant did not occur in resistant cells, such as a fibroblast cell line, MRC5, and HUVECs *in vitro*, suggesting that these results are cell type specific. In addition, by extending the linker between the monovalent halves of a bivalent IAP antagonist, the authors demonstrated loss of this unique property of bivalent IAP antagonists in a linker length-dependent manner. Although the exact mechanism for this effect is yet to be determined, these results provide evidence that, amongst the total cIAP1, TRAF2-associated cIAP1 is a distinct target of bivalent IAP antagonists only.

While the potential of small-molecule IAP antagonists for the treatment of cancer and other diseases was reported as early as 2004, the biochemical and biophysical characterization amongst and within these two distinct classes of therapeutic ligands, that is, monovalent and bivalent IAP antagonists, has received limited attention. The results by Mitsuuchi and colleagues, together with reports detailing the consequences of pan-IAP antagonism,^9, 10, 11^ suggest that a greater appreciation of these diverse ligands will be forthcoming. As the therapeutic potential of these agents are revealed in ongoing clinical trials, a re-examination of their biochemical properties, and the cell death and/or signaling pathways engaged following IAP antagonist treatment will lead to a more rational application of this important class of pharmacological agents.

## Figures and Tables

**Figure 1:**
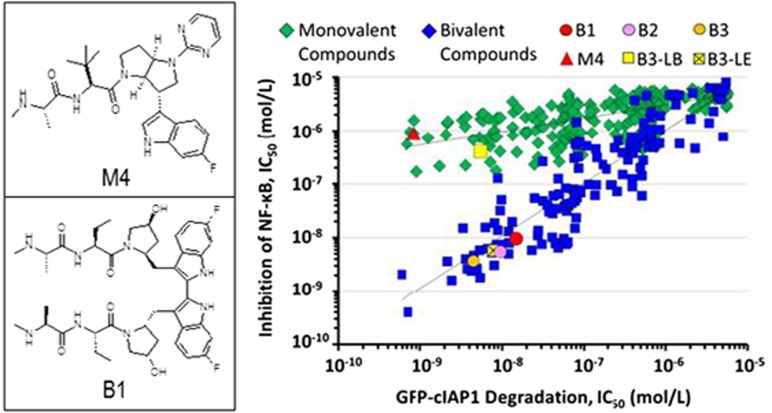
Monovalent and bivalent IAP antagonists differ in their abilities to inhibit NF-*κ*B signaling. Mitsuuchi *et al.*^13^ describe a key biochemical difference between monovalent and bivalent IAP antagonists exemplified by M4 and the clinical compound birinapant (B1), respectively. Despite their abilities to induce the degradation of endogenous or GFP-tagged cIAPs, bivalent IAP antagonists, such as B1, are superior to monovalent IAP antagonists in potently inhibiting TNFR1-dependent NF-*κ*B activation. The authors have attributed this property to the preferred ability of bivalent IAP antagonists to induce the efficient degradation of TRAF2-associated cIAPs
